# Progressive neurological disease induced by tacrolimus in a renal transplant recipient: Case presentation

**DOI:** 10.1186/1471-2369-7-7

**Published:** 2006-03-31

**Authors:** Marjan Chegounchi, Michael G Hanna, Guy H Neild

**Affiliations:** 1 Department of Nephrology, UCL Hospitals Trust, Middlesex Hospital, W1T 3AA, London; 2 Department of Neurology, UCL Hospitals Trust, Middlesex Hospital, W1T 3AA, London

## Abstract

**Background:**

Tacrolimus and cyclosporine, both calcineurin inhibitors, can cause neurological side effects. While mild symptoms such as tremor are well recognised, severe complications including seizures and encephalopathy are poorly documented following renal transplantation.

**Case presentation:**

We report a 42 year old man who received a cadaver renal transplant. He received tacrolimus and prednisolone. The course was uneventful for 6 weeks when he became intermittently confused, with unsteady gait and slurred speech. Following a grand mal convulsion he was admitted. He had no focal neurological signs, cerebrospinal fluid was normal; electroencephalogram was consistent with temporal lobe partial epilepsy. The magnetic resonance imaging of brain showed widespread changes with multiple areas of low signal intensity in brain stem and cerebral hemispheres. He was readmitted 3 weeks later after further fits, despite anti-convulsant therapy. He was psychotic with visual hallucinations, and rapidly became obtunded. Although his tacrolimus blood concentration had been kept in the normal range, his symptoms improved dramatically when the tacrolimus was stopped.

**Conclusion:**

Severe central nervous system toxicity from calcineurin inhibitors has been rarely reported in renal transplantation and we found only one report of tacrolimus-induced toxicity in an adult. We believe the condition is frequently undiagnosed. It is a very important diagnosis not to miss as the remedy is simple and failure may result in unnecessary brain biopsy, as well as irreversible injury.

## Background

Neurological complications of tacrolimus are usually mild (tremors, paraesthiae and myalgia), but can be severe with encephalopathy, seizures and coma. Severe complications have been more frequently reported following liver and lung than with renal transplantation [1-4], and typically occur with tacrolimus concentrations consistently above the therapeutic range of 15 ng/ml. We report the case of progressive neurological deterioration in a renal allograft recipient who suffered convulsions, intermittent confusion and finally mental obtundation. Severe central nervous system (CNS) toxicity from calcineurin inhibitors has been rarely reported in renal transplantation [2,5] and we found only one report of tacrolimus in an adult[6]. We believe the condition is frequently undiagnosed, and in our case the diagnosis was not considered for 2 months after onset of symptoms.

## Case report

A 42 years old man of mixed race with an African father had originally presented with severe hypertension and renal failure. Renal biopsy showed severe vascular pathology but no primary glomerular disease. He underwent a cadaver renal transplant (111 mis-match) in July 2002 and received tacrolimus and prednisolone. The kidney functioned immediately. On day 8 (d8) with a plasma creatinine that had not fallen below 180 μmol/l and tacrolimus concentration consistently 15 ng/ml (target concentration 10–15 ng/ml), he had a renal biopsy that showed acute rejection (Banff IIa). He received intravenous methylprednisolone and Cellcept 500 mg bd was added. The creatinine fell to125 μmol/l.

**Figure 1:**
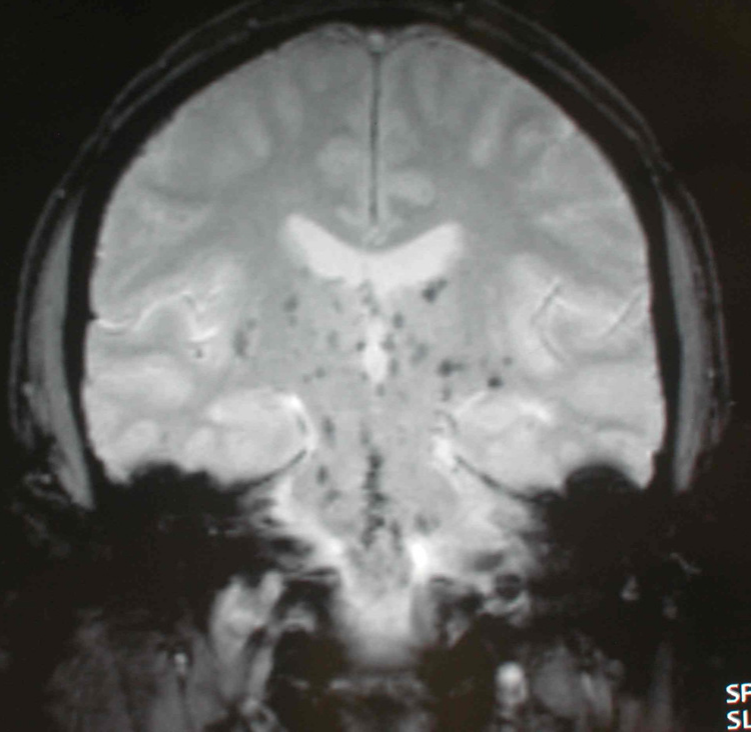
**MRI Brain**. There are multiple areas of low signal intensity in the pons, medulla oblongata, basal ganglia and also in the cerebral hemispheres (MRI Brain T1).

Eight weeks after transplantation (d59) he was admitted to another hospital following a road traffic accident. Whilst driving he had experienced sudden onset of drowsiness with headache and numbness in his fingers and toes. He temporarily lost consciousness and collided with another vehicle. Computed tomography (CT) scan of his head showed no abnormality and he was discharged home the following day. The following week he was readmitted (d66). He had a month history of persistent frontal headache, relieved by simple analgesic. He was intermittently confused and unable to recognize members of his family. This was associated with unsteady gait and slurred speech. During these episodes he appeared withdrawn and somewhat blank. Episodes lasted 1–8 hours. He had had one grand mal convulsion witnessed at home. There was no previous history or family history of neurological disease. On admission, he was afebrile with blood pressure of 120/75. He had no focal neurological deficit and no meningism. Cerebrospinal fluid (CSF) examination was normal: microscopy, Gram and Zeihl-Nielson stain, cytology, virology, glucose, protein, cultures and India-ink for cryptococcus were unremarkable. Blood cultures, blood count, Chest and skull X-ray were normal. Electroencephalogram (EEG) revealed sharpening and spikes independently in both temporal lobes consistent with temporal lobe partial epilepsy. The magnetic resonance imaging (MRI) of brain showed multiple areas of low signal intensity (T1-weighted FLASH) in the pons, medulla oblongata, basal ganglia and also in the cerebral hemispheres (figure 1). These lesions were reported as 'consistent with small vessel ischemia secondary to hypertension'. The diagnosis of partial epilepsy was made and he was started on lamotrigine. His renal function remained stable. Magnesium was consistently at the lower limit of normal range 0.6 mmol/l (normal range 0.6 – 1.1 mmol/l), cholesterol 4.5 mmol/l (2.3 – 5.2 mmol/l). The tacrolimus level was kept within range 8–15 ng/dl.

He was readmitted three weeks later (d113) having had further fits. He was agitated, psychotic with visual hallucinations. No focal neurology was found and he rapidly became obtunded with a Glasgow coma score of 6–8/15. Repeat CT and MRI were unchanged. Lumbar puncture revealed high open pressure and raised protein 1.3 g/l but CSF analysis was otherwise normal. Repeat EEG was consisted with complex partial status epileptics with "frequent frontal notched theta activity". He was treated with antibiotic and antifungal agents and phenytoin infusion. There was no improvement in his condition and a diagnosis of tacrolimus-induced neurotoxicity was made (tacrolimus 10 ng/ml). Tacrolimus was switched to cyclosporine. Over the next few days he showed rapid and progressive improvement. He recovered fully and was discharge home. He has had no further seizure activity nor neurological symptoms since tacrolimus had been stopped. He continues to do well. A MRI scan performed in 2004 no longer showed the earlier lesions.

## Conclusion

Severe calcineurin inhibitor-induced CNS toxicity is associated with a leucoencephalopathy [7]. Neuro-radiological abnormalities are most commonly seen in the parietal-occipital region and appear as multifocal low attenuation of white matter on CT scan with corresponding hyper-intense lesions on MRI (T2-weighted)[3]. The changes may be very subtle[8,9]. It has been suggested that the less frequent grey matter pathology can be visualised better using MRI with fluid-attenuated inversion recovery (FLAIR)[10]. These changes, however, are not specific. Sixteen patients with cyclosporine neurotoxicity investigated with MRI scans had findings identical to those reported in hypertensive encephalopathy and in 14 of 16 the symptoms improved after Bp became normal[11].

CSF analysis in calcineurin inhibitor-induced CNS toxicity is either normal or there may be a slight elevation of protein concentration as in our case[12]. EEG may be non-specifically encephalopathic with diffuse slowing or may shown frank epileptic discharges as in the present case[13].

Cases generally occur early when blood levels of tacrolimus or cyclosporine are high, but can occur with normal levels. A review of 50 reported cases found a median time to onset of 28 days, with 82% of cases occurring within 90 days [2]. Typically symptoms improved promptly when the dose was reduced or the drug stopped, but recovery can be incomplete [1,14,15]. The onset can be late, severe and irreversible [1,6].

From the review of 50 cases by Singh and colleagues[2], seizures 74%, altered mental status 50%, and visual abnormalities 28% were the most frequently presenting features. Ten percent of the patients had fever with no documented source of infection[2].

Severe neurotoxicity in renal patients is usually multifactorial, with patients systemically unwell, uraemic and receiving combinations of potentially neurotoxic drugs. With cyclosporine leucoencephalopathy other contributing factors have been reported. Hypocholesterolaemia[13] and hypomagnesaemia[16] have been implicated, but were not present in our case; similarly, onset has often been associated with high blood pressure and fluid overload [11], which were also not features of this case. Initially it was assumed that the encephalopathy had an ischaemic basis, but more recently the similarity to mitochondrial encephalopathy has been described and investigated [7].

In our case the tacrolimus levels were often in the high normal range, particularly in the second month of his illness which was the fourth transplant month. We would recommend that tacrolimus levels are kept below 10 ng/ml after the second transplant month in straightforward transplants. Calcineurin inhibitor-induced CNS toxicity is a very important diagnosis not to miss as the remedy is simple and failure may result in unnecessary brain biopsy, as well as irreversible injury.

## Competing interests

The author(s) declare that they have no competing interests.

## Authors' contributions

All the authors were involved in the clinical management of the case and the writing of the report.

## Pre-publication history

The pre-publication history for this paper can be accessed here:


